# Progress in Orthopædic Surgery

**Published:** 1903-09-05

**Authors:** 


### PROGRESS IN ORTHOPÆDIC SURGERY.

**The Surgery of the Paralyses of Children.**—Robert Jones[1] has given an instructive address on this subject, in which he deals with infantile paralysis and spastic paralysis. In infantile paralysis he urges that the aim of the surgeon should be to prevent deformity, as well as to cure it, when it has supervened. The factors in the production of deformity are gravity, body-weight, the shape of the articular facets, and unbalanced muscular action ; and he illustrates his proposition by concrete examples in the cases of the foot, the knee, and the hip. He further thinks that surgery has been too long silenced by the pathological reports of these cases, and that we are too ready to infer that because some of the motor cells are damaged, the muscles, either as groups or entities, are hopelessly destroyed. Extended clinical experience proves that this is not so, for muscles which are apparently entirely paralysed will recover often to a very considerable degree when placed in suitable conditions. And this fact is the keynote of the surgical treatment of infantile paralysis. It is best illustrated by dropped wrist from paralysis of the extensors. Recovery will never take place so long as the weakened extensors are continuously stretched by allowing the wrist to droop. If the hand is kept hyper-extended, and the extensor muscles are suitably massaged, recovery soon begins to be manifest, and will be complete in most cases in 18 months to two years. By this treatment the lengthened extensor muscles are shortened, and the contracted flexors are lengthened, and thus the muscular balance is restored. Tendon transplantation is of the utmost value in the treatment of paralytic deformities, because it is scientific in its methods and easy and safe in its application. It is scientific because it aims at distributing around a joint in an equal manner the residue of muscular power. Or to put the matter more clearly, and to take a definite example : if the foot is in a position of equino-valgus it is quite evident that the tendo Achillis and peronei are exerting an undue power, and that this power is running to waste, while the invertors and dorsiflexors are unable to oppose them. The scientific method of transplantation will transfer part of the force in the tendo Achillis to the invertors, and part of the power of the evertors into the invertors, thus obtaining good muscular balance around the ankle. This is a more rational proceeding than the old practice of dividing the tendons of the healthy muscles and then trying to maintain the foot in good position by apparatus, which must be worn for a life-time. In the treatment of spastic paralysis equally great advances have been made. Every contracted tendon and muscle should be divided, the limbs straightened, and all flexor deformities fully overcome, and then, this being highly important, the child must be educated to use his limbs properly.

**Tendon Transplantation for Infantile Paralysis.**—W. P. Montgomery[2] gives accounts of 25 cases in which this operation was done to remedy the sequelæ of anterior poliomyelitis. After reviewing the history of the operation, Montgomery details four classical methods, viz.: (1) Section of the tendon of a healthy muscle and its implantation into the tendon of a paralysed one; (2) the "passive" method of Hoffa, which consists in section of the tendon of a paralysed muscle and its attachment to an acting undivided muscle; (3) the transference of a slip from an active muscle tendon to a paralysed one ("active" method of Hoffa); (4) the attachment of an active muscle or tendon, or of a slip from it, to the periosteum at a new site. Montgomery has employed all these methods in his cases. There are at least nine other methods in vogue, to which allusion might well have been made. Certain conclusions are deduced from the cases. These are briefly: (1) If the acting and paralysed muscles are the more nearly allied in their actions, the improvement is much more rapid and the earlier the results will appear; (2) the greater the length of the tendon of a paralysed muscle to which the acting muscle is applied, the less satisfactory the earlier result and the slower the improvement will be; this is due to stretching of the paralysed tendon; (3) the results of the passive method, division of the paralysed tendon and its suture to the intact acting one are much inferior to those of the active method, in which a slip is taken from the normal tendon; (4) there is no doubt that at the time of transplantation all deformity must be corrected; (5) as regards after-treatment, the ideal method is to begin massage and passive movements after three weeks and gentle active movements after four weeks. The material used for tendon grafting was silk, and in no cases did any stitch abscesses arise.

**Snap or Trigger Finger.**—H. L. Barnard[3] records four cases of this interesting and not uncommon condition. In one case on moving the finger painful nodules could be felt slipping up and down, and these nodules were in the flexor sublimis tendon; and in another instance where the thumb was affected the nodules were on the flexor longus pollicis tendon, and in two other cases painful nodules could be felt wedged in between the sesamoid lines of the short flexor of the thumb. It is pointed out that the majority of cases have occurred in women, and most of them are over 14 years of age. The snapping is due to changes in the tendon, in the tendon sheath, and in the joint which snaps. As to treatment, many of the cases are evanescent; and for such, rests on a splint with pressure from a pad placed over the swelling, and later on massage, appear to be suitable and successful forms of treatment. But if a nodule can be felt and it persists for some time, the right course to take is to expose the tendon and remove the nodule or lessen the disproportion between the tendon and its sheath. If synovial fringes exist they should be removed. F. Griffith[4] also describes a case, which affected the middle and ring fingers in a woman. In an analysis of 121 cases, Necker found either acute or chronic rheumatism in 52; traumatism in 13; occupation causes in 47; congenital conditions in two cases, and in seven no cause could be assigned. Griffith is in accord with Barnard as to the causation and treatment. Whiteford[5] records a case in a man aged 39 who had become an enthusiast at golf. The left little finger was affected. With precautionary measures the affection subsided and operation was unnecessary.

**Forcible Rectification of the Spine for Pott's Disease.**—J. V. Young[6] describes a case under his treatment at Beyrout in Syria, and alludes to the work of Tubby and Jones[7] in this direction. Young followed their procedure almost exactly. He found the operation safe and easy, and was pleased with the immediate result. Now that some years have elapsed since this treatment was commenced, there is no doubt that if cases be properly cared for afterwards there is lasting improvement.

[1] Lancet, Feb. 14, 1903, p. 422. [2] Med. Chron., November, 1902, p. 97. [3] Practitioner, February 1903, p. 178. [4] Anns. of Surg., October 1902, p. 586. [5] Brit. Med. Jour., Jan. 31, 1903, p. 251. [6] New York Med. Rec., August 1902, p. 173. [7] Med. Annual, 1898, and Clin. Soc. Trans., 1899 and 1901.

